# Prediction of Lumbar Drainage-Related Meningitis Based on Supervised Machine Learning Algorithms

**DOI:** 10.3389/fpubh.2022.910479

**Published:** 2022-06-28

**Authors:** Peng Wang, Shuwen Cheng, Yaxin Li, Li Liu, Jia Liu, Qiang Zhao, Shuang Luo

**Affiliations:** ^1^Department of Neurosurgery, Cancer Prevention and Treatment Institute of Chengdu, Chengdu Fifth People's Hospital (The Second Clinical Medical College, Affiliated Fifth People's Hospital of Chengdu University of Traditional Chinese Medicine), Chengdu, China; ^2^West China Fourth Hospital/West China School of Public Health, Sichuan University, Chengdu, China

**Keywords:** lumbar drainage, meningitis, machine learning, prediction model, infectious diseases

## Abstract

**Background:**

Lumbar drainage is widely used in the clinic; however, forecasting lumbar drainage-related meningitis (LDRM) is limited. We aimed to establish prediction models using supervised machine learning (ML) algorithms.

**Methods:**

We utilized a cohort of 273 eligible lumbar drainage cases. Data were preprocessed and split into training and testing sets. Optimal hyper-parameters were archived by 10-fold cross-validation and grid search. The support vector machine (SVM), random forest (RF), and artificial neural network (ANN) were adopted for model training. The area under the operating characteristic curve (AUROC) and precision-recall curve (AUPRC), true positive ratio (TPR), true negative ratio (TNR), specificity, sensitivity, accuracy, and kappa coefficient were used for model evaluation. All trained models were internally validated. The importance of features was also analyzed.

**Results:**

In the training set, all the models had AUROC exceeding 0.8. SVM and the RF models had an AUPRC of more than 0.6, but the ANN model had an unexpectedly low AUPRC (0.380). The RF and ANN models revealed similar TPR, whereas the ANN model had a higher TNR and demonstrated better specificity, sensitivity, accuracy, and kappa efficiency. In the testing set, most performance indicators of established models decreased. However, the RF and AVM models maintained adequate AUROC (0.828 vs. 0.719) and AUPRC (0.413 vs. 0.520), and the RF model also had better TPR, specificity, sensitivity, accuracy, and kappa efficiency. Site leakage showed the most considerable mean decrease in accuracy.

**Conclusions:**

The RF and SVM models could predict LDRM, in which the RF model owned the best performance, and site leakage was the most meaningful predictor.

## Highlights

- The supervised machine learning algorithm has value in developing the LDRM model.
- The RF and SVM models had acceptable performance.
- Site leakage was the most meaningful predictor.
- Our proposed model may serve as a decision-making tool in the clinic.

## Introduction

Lumbar drainage (LD) is a temporary technique for neurologically disease patients with the purpose of therapeutically or prophylactically releasing cerebrospinal fluid (CSF) and modulating CSF pressure (1, 2). This technique is conducted in isolation from the environment through a closed medical instrument with the catheter tip placed into the lumbar cistern. As a routine operation, it is widely used in clinical practice and manipulated by multiple specialists, including neurosurgeons, neurocritical care physicians, interventional radiologists, anesthesiologists, and otolaryngologists (3). The common LD indications consist of intraventricular or subarachnoid hemorrhage, CSF leakage, communicating hydrocephalus, and drainage of CSF during operation to increase anatomical exposure (4–8).

Previous studies reveal that LD can bring obvious clinical benefits to patients, such as reducing angiographic and symptomatic vasospasm, preventing permanent shunt dependency, accelerating CSF leakage healing, and predicting the outcome of hydrocephalus shunt (9–12). In addition, LD may be a safe and effective method to lower intracranial pressure in traumatic brain injury patients with refractory intracranial hypertension (13). However, the risks from LD are frequent and need to be noticed, although LD placement is often viewed as benign (2). One of the most common complications is lumbar drainage-related meningitis (LDRM), which has diverse incidence in different reports, usually as 3–20%, and a few may be as high as 40% (14). This complication can prolong the hospital stay, increase medical expenses, and even lead to catastrophic outcomes (15, 16).

Several risk factors have been identified to promote the occurrence of LDRM. These factors include duration days, drain opening, site leakage, admission to intensive care unit (ICU), diabetes, and accompanied craniotomy (15–18). In our earlier study, we proposed a prediction model by screening the risk factors of LDRM and established a nomogram as a simple tool to estimate the infection risk (19). However, this model was built based on a traditional logistic regression method, which is challenging to fit the actual distribution of data and deal with the problem of collinearity.

Novel supervised machine learning (ML) algorithms have become widely accepted in recent decades, and have emerged as a popular method of clinical infection research (20). The algorithms can build complex non-linear models that associate the independent features with dependent corresponds in large data sets, with high efficiency and accuracy (21). In this work, we used three frequent ML algorithms, the support vector machine (SVM), random forest (RF), and artificial neural network (ANN), to build prediction models of LDRM. We also evaluated the model performance and conducted internal validation to assess possible clinical application value.

## Methods

### Program Environment

The data preprocessing and model development in this research were implemented within the environment of R (4.1.2).

### Study Population

**Figure 1:**
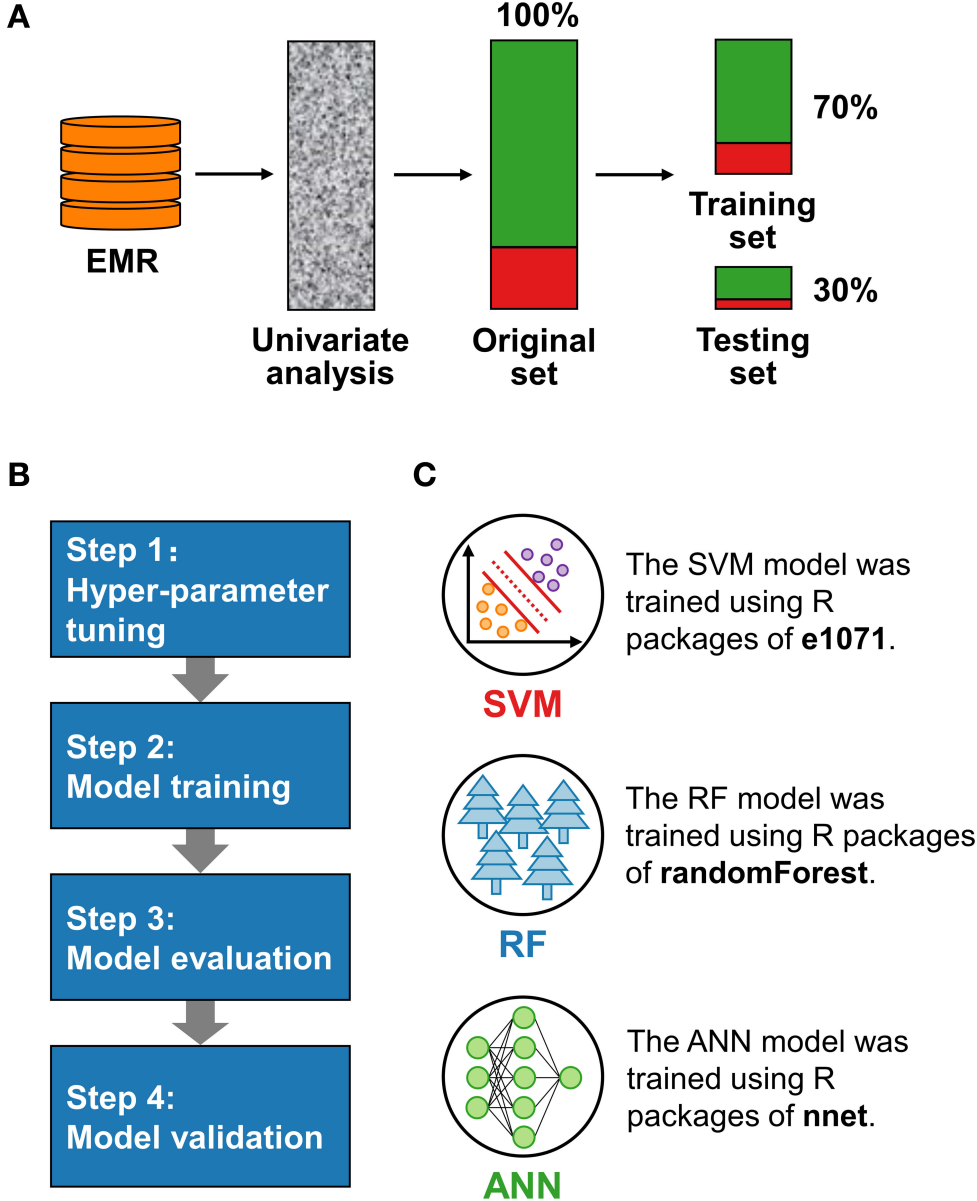
Schematic diagrams of data processing and model development. **(A)** The data from electronic medical records (EMR) were selected by univariate analysis to form an original set, which was divided into training and test sets by stratified sampling. **(B)** Models were developed through a procedure with four steps. **(C)** Three machine learning algorithms, the support vector machine (SVM), random forest (RF), and artificial neural network (ANN), were used for model training.

We used a cohort of 273 eligible cases, as described in our previous report (19). All the enrolled patients received LD treatment during a research period from January 2012 to December 2018 in the Chengdu Fifth People's Hospital (Chengdu, China). The original clinical features were extracted from the hospital electronic medical records, including sex, age, admission diagnosis, admission to surgery intensive care unit (SICU), initial Glasgow coma scale score, blood CSF, malignancy, immunosuppression, diabetes, duration days, site leakage of CSF, accompanied craniotomy, and antibiotic treatment for other types of infection (before or after LD initiation). LDRM was identified as the response variable, in which meningitis was the positive response, and the contrary situation (without meningitis) was the negative response. After univariate analysis, we only collected statistically significant features related to the occurrence of LDRM to form the original set (Figure 1A). All procedures in this study were following the ethical standards of the institutional ethical committee of the Chengdu Fifth People's Hospital (ref. no. 2019–074), and with the 1964 Helsinki Declaration and its later amendments or comparable ethical standards.

### Data Pre-processing

All data had normalization (range “0–1”) to eliminate the dimensional influence between features and make different predictors comparable. We conducted stratified sampling according to the response, taking 70% of the original set as the training set and 30% as the testing set (Figure 1A). Compared with random sampling, stratified sampling can help achieve a consistent distribution of response in training and testing sets. Features were modified to categorical variables using *as.factor* function if they were automatically identified as numeric in R.

### Model Development

We selected three supervised ML algorithms, SVM, RF, and ANN, to construct binary LDRM prediction models following a procedure with four steps (Figure 1B).

(1) Hyper-parameter tuning. We adopted a 10-fold cross-validation and grid search to achieve the best parameters with minimal classification error for each algorithm. The *tune* function in R implemented this process.

(2) Model training. We trained models via three ML algorithms using the determined hyper-parameters. The SVM, RF, and ANN algorithms were proceeded by R packages of e1071 (22), randomForest (23), and nnet (24), respectively (Figure 1C). We also analyzed the importance of features in the RF model.

(3) Model evaluation. We drew the receiver operating characteristic curve (ROC) and precision-recall curve (PRC) and calculated the area under the two curves (AUROC and AUPRC) to evaluate the performance of different models. We also constructed the confusion matrix and calculated other performance indicators for evaluation, such as true positive ratio (TPR), true negative ratio (TNR), specificity, sensitivity, accuracy, and kappa coefficient.

(4) Model validation. We verified the trained models in the testing set for internal validation to determine whether the models were generalizable. Similarly, we computed performance indicators of the model in the testing set as the method in step three.

## Results

The original set of this study enrolled 273 LD patients, including 37 (13.6%) cases with meningitis and 236 (86.4%) without meningitis. The demographic information and univariate analysis of the data set are demonstrated in our previous report (19). Five features (admission to SICU, diabetes, duration days, site leakage, and associated craniotomy) had significant differences between infected and non-infected cases. See reference 19 for further details. We adopted stratified random sampling to divide the original set into training and testing sets. The former has 192 patients (70.3%) and the latter has 81 (29.7%). The distribution of LD patients with and without meningitis in different data sets is shown in Table 1.

**Table 1:** Distribution of lumbar drainage patients with and without meningitis in different data sets.

**Data Sets**	**Meningitis**	**Non-meningitis**	**Total**
Original set	37 (13.6%)	236 (86.4%)	273 (100%)
Training set	25 (13.0%)	167 (87.0%)	192 (100%)
Testing set	12 (14.8%)	69 (85.2%)	81 (100%)

**Figure 2:**
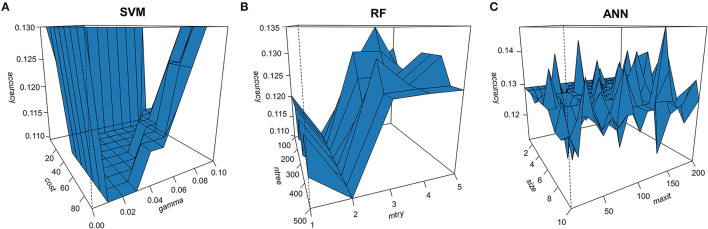
Perspective view of the outcomes in hyper-parameter tuning. The 10-fold cross-validation and grid search were used to obtain optimal hyper-parameters for three machine learning models. **(A)** The constraints violation cost (*cost*) and gamma parameter (*gamma*) in the support vector machine (SVM) model. **(B)** The number of trees to grow (*ntree*) and variables randomly sampled as candidates at each split (*mtry*) in the random forest (RF) model. **(C)** The number of hidden units (*size*) and the maximum number of iterations (*maxit*) in the artificial neural network (ANN) model.

We used the 10-fold cross-validation and grid search to obtain optimal hyper-parameters (Figure 2). The optimal constraints violation cost (*cost*) and gamma parameter (*gamma*) of the SVM model were 31 and 0.01, with a minimal error of 0.109. The optimal number of trees to grow (*ntree*) of the RF model was 500, and the number of variables randomly sampled as candidates at each split (*mtry*) was 2, with a minimal error of 0.110. As for the ANN model, the optimal number of units in the hidden layer (*size*) was 8, and the best maximum number of iterations (*maxit*) was 170, with a minimal error of 0.111.

**Figure 3:**
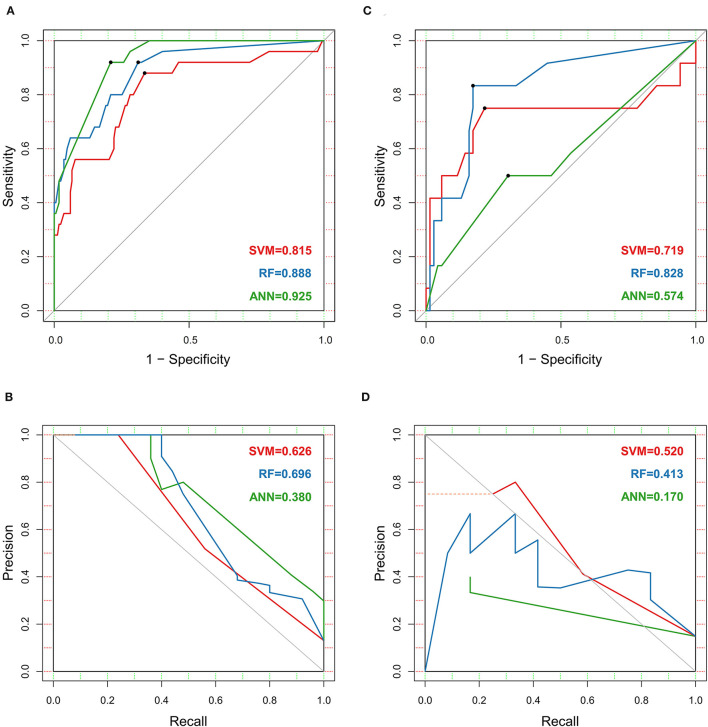
The area under the receiver operating characteristic curve (AUROC) and precision-recall curve (AUPRC) of three machine learning models, which were trained using the support vector machine (SVM), random forest (RF), and artificial neural network (ANN) algorithms. **(A)** AUROC of the training set. **(B)** AUPRC of the training set. **(C)** AUROC of the testing set. **(D)** AUPRC of the testing set.

**Figure 4:**
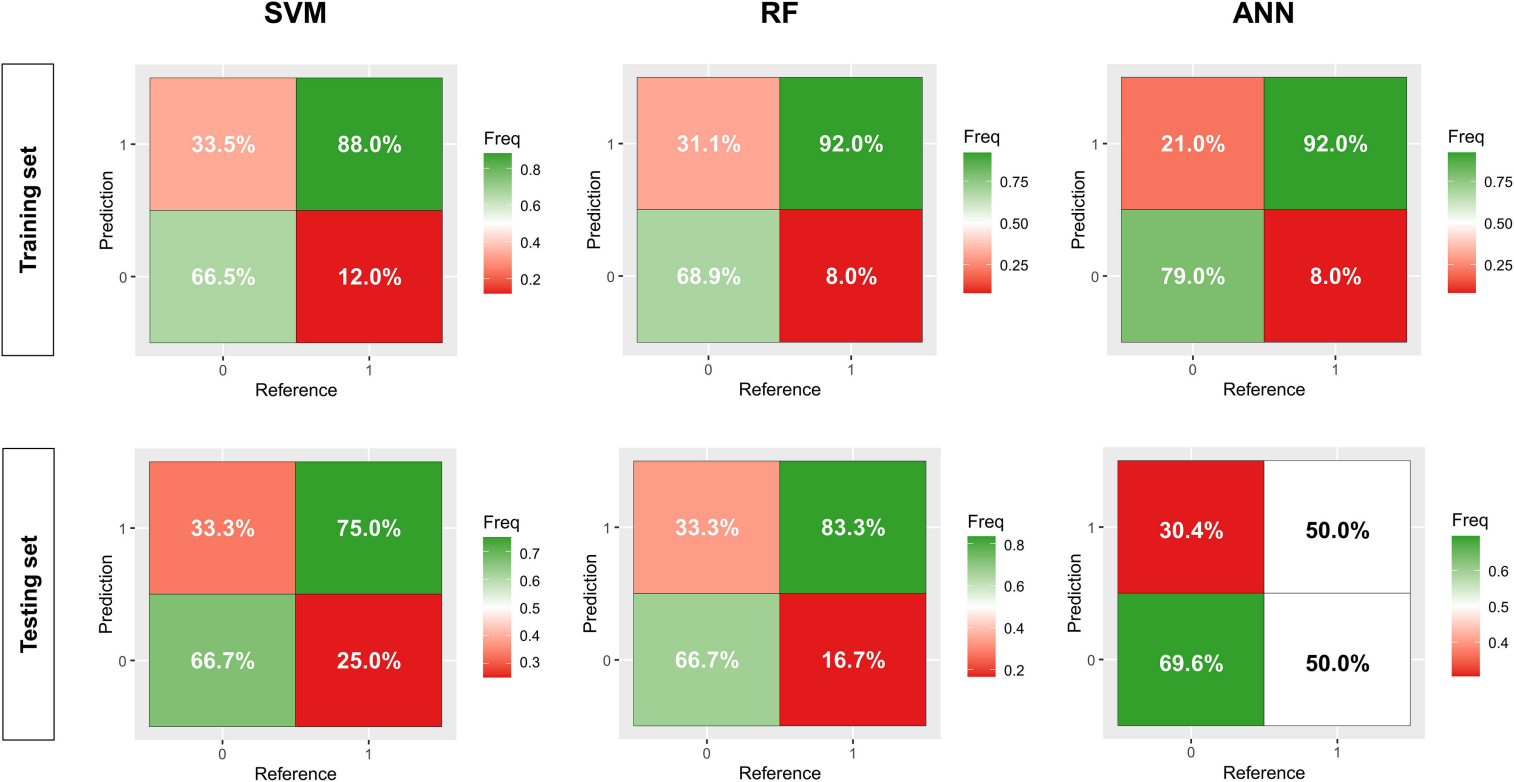
The confusion matrix of three machine learning models, constructed by the support vector machine (SVM), random forest (RF), and artificial neural network (ANN), respectively. The value in the upper-right grid represents the true positive ratio, and the value in the lower-left grid means the true negative ratio.

We established three ML models in the training set. The kernel used in the SVM model was *radial*. The AUROC of all three models exceeded 0.8; the ANN model had a maximal under curve area of 0.925 (Figure 3A). The SVM and RF models had acceptable AUPRC, both of which were more than 0.6. However, the ANN model had a low AUPRC, and the value was only 0.380 (Figure 3B). The RF and ANN models revealed similar TPR, while ANN had a higher TNR (Figure 4). And the ANN model showed better specificity, sensitivity, accuracy, and kappa efficiency (Table 2).

**Table 2:** Performance indicators of confusion matrix in training and testing sets.

**Models**	**Training set**				**Testing set**			
	**Specificity**	**Sensitivity**	**Accuracy**	**Kappa coefficient**	**Specificity**	**Sensitivity**	**Accuracy**	**Kappa coefficient**
SVM	0.665	0.880	0.693	0.287	0.783	0.750	0.679	0.247
RF	0.689	0.920	0.719	0.329	0.826	0.833	0.691	0.290
ANN	0.790	0.920	0.807	0.455	0.696	0.500	0.667	0.129

*SVM, support vector machine; RF, random forest; ANN, artificial neural network*.

Ulteriorly, we internally validated the established models in the testing set. The RF and SVM models maintained adequate AUROC (0.828 vs. 0.719), whereas the ANN models decreased by a prodigious degree (0.574) (Figure 3C). All three models had different levels of decrements in AUPRC, in which the RF and SVM models comparatively performed better (0.413 vs. 0.520) (Figure 3D). The RF model had better TPR, although the indicator of all models decreased comprehensively. And the TNR of SVM and RF models changed slightly, compared with a notable decline in the ANN model (Figure 4). RF showed better specificity, sensitivity, accuracy, and kappa efficiency than the other models (Table 2).

**Figure 5:**
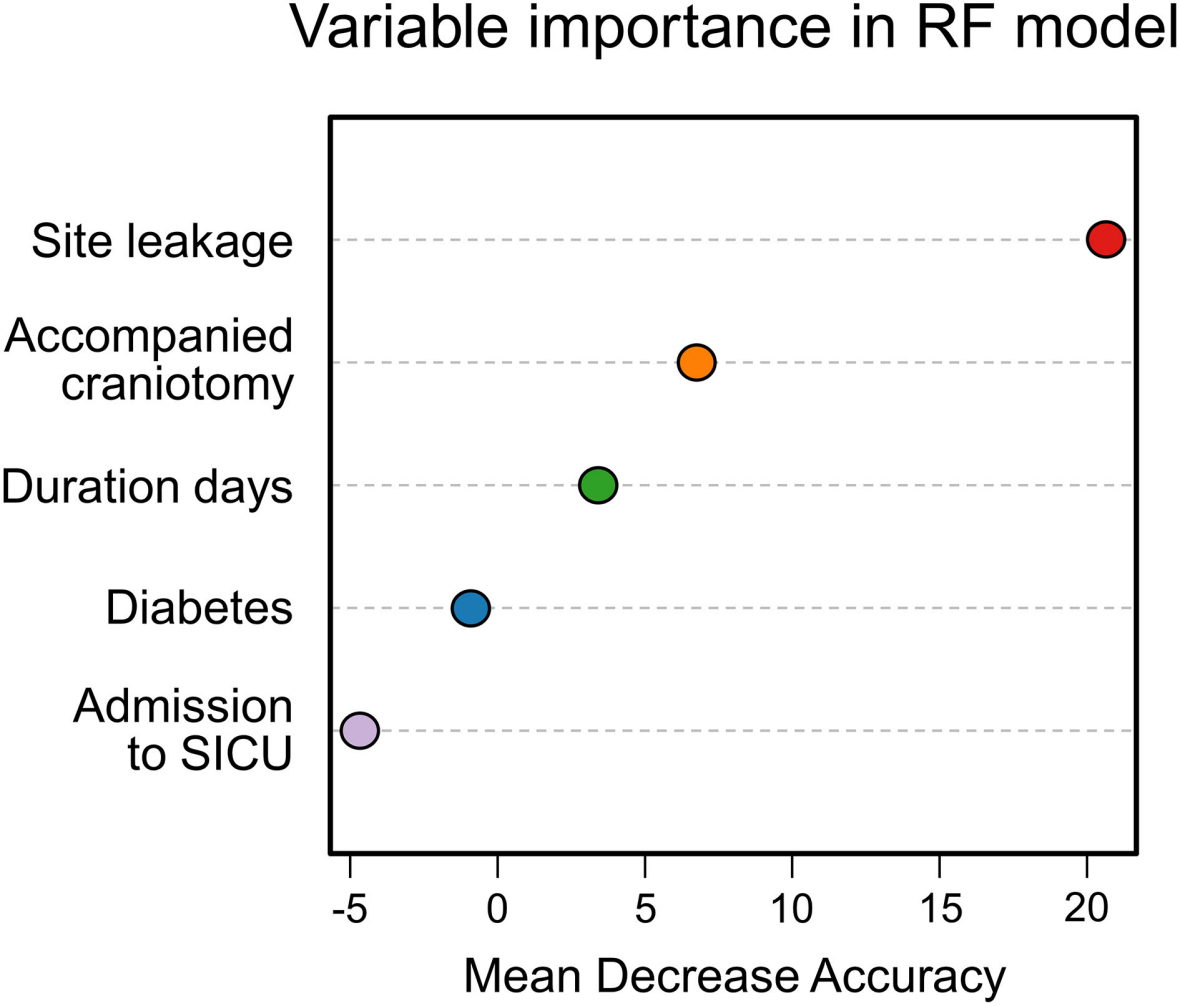
The importance of features in the random forest (RF) model. Site leakage showed the most considerable mean decrease in accuracy.

In addition, the importance of features was analyzed in the RF model, in which site leakage had a significant impact on the prediction accuracy, with the most meaningful mean decrease accuracy (Figure 5).

## Discussion

At present, scholars have an increasing enthusiasm for utilizing supervised ML to predict the occurrence of infection, including iatrogenic and non-iatrogenic (21, 25–33). Nonetheless, the study that forecasts the risk of LDRM in an early stage before the clinical diagnosis is limited, although we have proposed a prediction model using the traditional logistic regression algorithm (19). To search for potential ML models and improve the prediction accuracy, we used three prevalent ML algorithms to establish the LDRM prediction models in this research. The main findings are the RF and SVM models showed the ability to predict LDRM, in which the RF model had the best performance among all established models, and site leakage was the most meaningful predictor.

Data preprocessing is an important preceding step to initiating machine learning (34). The features included in the model often have different dimensions and units, which will affect the results of data analysis and cause bias (35). We normalized the values (range “0–1”) between included features to eliminate the overrepresentation or underrepresentation between predictors. In addition, we conducted feature selection to delete redundant or useless characteristics and retain the contributing variables in the prediction (29, 35). We used the univariate analysis consistent with our previous studies to make the included variables concordant so that the models are comparable.

We included five features as model predictor variables, which were significantly different in infected and non-infected groups in the this study. Duration days and site leakage are two features directly related to LD. Although the relationship between drainage time and infection is non-linear (36), it is difficult to obtain an infection cutoff as the risk increases gradually and progressively (16). Site leakage is another significant predictor variable, regarded as a critical driving factor causing retrograde infection (19). Diabetes is usually a risk factor for surgical site infection because diabetic patients are prone to hyperglycemia, vascular changes, and white blood cell dysfunction (37). Furthermore, admission to SICU and associated craniotomy are predictors included in this work, with the possible reason these LD patients are more severe and susceptible to bacteria.

Typically, the original set is divided into a large set (training set) for model training and a small set (testing set) for model validation. Some studies that predict infection use random sampling to split the original data (25, 27). Considering that this method may cause a disproportionate distribution between different data sets, we used stratified sampling in the this study. We also recommend this hierarchical data segmentation method, of which the utilization can contribute to balancing the class distributions within the splits. Data imbalance in ML algorithms may be an obstacle to obtaining excellent results (38). Some data resampling methods, such as oversampling or under sampling, are likely to help solve this problem. However, we did not resample data because the benefit of data balance is limited in infection prediction, given the low rate of positive events (25).

Hyper-parameter has a considerable influence on the model performance in ML. These parameters are predetermined but not obtained through the training process (39). It is needed to optimize the hyper-parameters for the ML models to improve their performance. Improper selection of hyper-parameters in some models, such as SVM and ANN, can significantly affect the outcome (35). In ML, a validation set is usually established, with the role of adjusting hyper-parameters. Nevertheless, we did not divide a validation set separately in the this study due to the limited data. We, instead, adopted a 10-fold cross-validation to achieve the optimal hyper-parameter in the training set, using the *tune* function of R. This general function uses grid search to adjust the hyper-parameter of the ML methods within the provided parameter range.

We established prediction models of LDRM using three ML algorithms. The model evaluation in the training set showed that the ANN model had excellent AUROC and additional performance indicators, including TNR, specificity, sensitivity, accuracy, and kappa efficiency. However, this model had a minimal area under the PRC, suggesting that it may not be satisfying because the PRC is more informative than ROC when dealing with imbalanced data sets (40). This conclusion is further confirmed when using the testing set for model verification. The performance of the ANN model decreased notably, which reveals there might be overfitting during training. Some previous studies also support our viewpoint, in which the ANN model does not achieve the best performance in infection prediction (27, 32).

On the contrary, the RF model in this study showed encouraging performance both in the training and testing sets. This model also outperforms our earlier logistic regression model (AUROC 0.888 vs. 0.837), and it consequently may be the most promising ML model for predicting LDRM. In addition, the strongest predictor of LDMR in the RF model was site leakage, which coincides with our previous studies. We have proposed a possible reason that the site leakage of LD is usually inconspicuous to be found, resulting in retrograde infection through CSF or infected soft tissue (19). Another model based on the SVM algorithm also had acceptable performance, although indicators were not superior to the RF model except AUPRC in the testing set. With further optimization, this model may become an alternative candidate for predicting LDMR.

Although the effective prevention of LDRM needs additional research, the ML models in this study, for example, may play a meaningful role. These prediction models can help clinicians and nurses judge the possibility of LDRM and identify high-risk patients when multiple risk factors coexist, to strengthen monitoring or adjust treatment strategy (30). Some procedures that increase the infection risk, such as CSF sampling, can be accurately enhanced or diminished as appropriate. Moreover, early prediction of LDRM may improve clinical outcomes and reduce medical costs, as the infection is closely related to disease deterioration and extra drug use (15, 17). It is worth noting that optimizing the model according to the target population is encouraged, given that the patient population, clinical scheme, and possible risk factors are diverse among institutions (26).

This study is the first to predict LDRM using supervised ML algorithms, in which we constructed and evaluated three prevalent models. However, there were several limitations. (1) Our study included retrospective data from a single research center to establish ML models, which may result in selection bias and introduce uncontrollable confounding factors. (2) We only used three ML algorithms, and other unused algorithms may help achieve models with better prediction performance. We also did not use unstructured data, which may contain extra prediction information (31). Furthermore, the modeling method we used entirely depends on supervised ML, which requires a lot of expensive and time-consuming tag data and may not extend well to related but non-identical tasks. (3) We did not conduct external verification of the established models, although some of them had a good performance during internal verification. It is necessary to update the model and verify the generalization in other clinical environments. In addition, whether these models can improve the clinical prognosis of LD patients and reduce medical costs remains to be explored.

## Conclusion

In summary, this study constructed and verified three supervised ML prediction models to predict LDRM. The results suggest that RF and SVM models had the predictive power, in which the RF model owned the best performance, and site leakage was the most meaningful predictor. Our research highlights that the prediction model based on the ML algorithm, with further optimization, may become an important decision-making tool for clinical staff in the future, supplementing the existing schemes to identify high-risk patients.

## Data Availability Statement

The raw data supporting the conclusions of this article will be made available by the authors, without undue reservation.

## Ethics Statement

The studies involving human participants were reviewed and approved by Ethical Committee of Chengdu Fifth People's Hospital. Written informed consent to participate in this study was provided by the participants' legal guardian/next of kin.

## Author Contributions

PW: conceptualization, funding acquisition, and writing—original draft. SC: methodology, visualization, and supervision. YL: formal analysis. LL, JL, and QZ: data curation and investigation. SL: validation and writing—review and editing. All authors contributed to the article and approved the submitted version.

## Funding

This research was supported by grants from the Xinglin Scholar Discipline Talents Scientific Research Promotion Plan of Chengdu University of TCM (YYZX2021047), the Chengdu High-level Key Clinical Specialty Construction Project (GSPZX2021-15), and the Foundation of Sichuan Health Commission (19PJ016).

## Conflict of Interest

The authors declare that the research was conducted in the absence of any commercial or financial relationships that could be construed as a potential conflict of interest.

## Publisher's Note

All claims expressed in this article are solely those of the authors and do not necessarily represent those of their affiliated organizations, or those of the publisher, the editors and the reviewers. Any product that may be evaluated in this article, or claim that may be made by its manufacturer, is not guaranteed or endorsed by the publisher.

## Abbreviations

LD: Lumbar drainage
CSF: Cerebrospinal fluid
LDRM: Lumbar drainage-related meningitis
ICU: Intensive care unit
ML: Machine learning
SVM: Support vector machine
RF: Random forest
ANN: Artificial neural network
SICU: Surgery intensive care unit
ROC: Receiver operating characteristic curve
PRC: Precision-recall curve
AUROC: Area under receiver operating characteristic curve
AUPRC: Area under precision-recall curve
TPR: True positive ratio
TNR: True negative ratio.
